# Trusted Sources of COVID-19 Vaccine Information among Hesitant Adopters in the United States

**DOI:** 10.3390/vaccines9121418

**Published:** 2021-12-01

**Authors:** Rachel S. Purvis, Emily Hallgren, Ramey A. Moore, Don E. Willis, Spencer Hall, Morgan Gurel-Headley, Pearl A. McElfish

**Affiliations:** 1 College of Medicine, University of Arkansas for Medical Sciences Northwest, 1125 N. College Avenue, Fayetteville, AR 72703, USA; rspurvis@uams.edu (R.S.P.); ehallgren@uams.edu (E.H.); rameymoore@uams.edu (R.A.M.); dewillis@uams.edu (D.E.W.); 2 Office of Community Health and Research, University of Arkansas for Medical Sciences Northwest, 1125 N. College Avenue, Fayetteville, AR 72703, USA; shall@uams.edu; 3 College of Medicine, University of Arkansas for Medical Sciences, 4301 W. Markham Street, Little Rock, AR 72205, USA; mpgurel@uams.edu; 4 Fay W. Boozman College of Public Health, University of Arkansas for Medical Sciences, 4301 W. Markham Street, Little Rock, AR 72205, USA

**Keywords:** COVID-19, COVID-19 vaccine, vaccine hesitancy, trusted sources

## Abstract

The World Health Organization has identified vaccine hesitancy as a top health concern. Emerging research shows that those who are hesitant may still get vaccinated; however, little is known about those who say they are hesitant but still get vaccinated. Most people have high trust in several sources of COVID-19 information, and trust in certain information sources such as the Centers for Disease Control and Prevention and health care providers was associated with being vaccinated. This study explored trusted information sources among hesitant adopters in the United States with a survey respondents completed while waiting after receiving a COVID-19 vaccine dose. The study included (*n* = 867) respondents. The majority of respondents were female (60.21%); were between the ages of 18 and 44 years old (71.97%); and were diverse, with most identifying as White (44.54%) or Hispanic/Latinx (32.55%). Hesitant adopters reported multiple trusted sources of COVID-19 vaccine information, which can be grouped into four emergent subthemes: (1) Health care/Medical science, (2) Personal relationships, (3) News and social media, and (4) Individual/Myself. Some respondents expressed a distrust of all sources of COVID-19 vaccine information, despite receiving the vaccine, describing a lack of trust in traditional sources of information such as the mainstream media or government. This study contributes to the literature by documenting trusted sources of COVID-19 vaccine information among hesitant adopters in the United States. Findings provide important insights about respondents’ trusted sources of COVID-19 vaccine information that can inform future public health messaging campaigns intended to increase vaccine uptake among hesitant adopters.

## 1. Introduction

The Centers for Disease Control and Prevention (CDC) reported the first laboratory-confirmed case of COVID-19 in the United States (US) in January 2020 [1], and since that time, more than 37,768,911 cases of COVD-19 have been documented in the US [2]. COVID-19 vaccinations began in December 2020 [3], with high demand as early adopters received the vaccine. However, demand slowed [4], and as of August 2021, only 51.5% of the US is vaccinated [5]. In contrast, the United Arab Emirates and Portugal currently have almost 80% of their populations vaccinated [6]. Low vaccination rates are often attributed to vaccine hesitancy, with minority communities having the highest levels of vaccine hesitancy and the lowest vaccination rates [7].

The World Health Organization has identified vaccine hesitancy as a top health concern [8]. Vaccine hesitancy is often described as a behavior ranging from refusing to be vaccinated to delaying vaccination [9]; however, other scholars see vaccine hesitancy as an attitude that is related to but not synonymous with vaccine behavior [10,11]. Emerging research shows that those who are hesitant may still get vaccinated [12]. Very little is known about those who say they are hesitant but still get vaccinated [13], leaving many questions about “hesitant adopters” unanswered. 

Most people have high trust in several sources of COVID-19 information [14]. Furthermore, trust in certain information sources such as the CDC and health care providers was associated with being vaccinated, and those reporting high trust in these sources were more likely to encourage family members and less likely to discourage friends from being vaccinated [14]. Individuals who receive information via traditional media sources such as national television, national newspapers, and local newspapers were most likely to accept the COVID-19 vaccine [15]. US college students reported that trusted information sources play a critical role in predicting vaccine acceptance [16].

No studies have documented the trusted sources of information specifically among hesitant adopters as a distinct population. To encourage uptake among the vaccine-hesitant, it is important to identify their most trusted sources of information about the COVID-19 vaccine. This study conducted a mixed-methods examination of trusted sources of information among hesitant adopters in the US.

## 2. Methods

### 2.1. Study Design, Aims, and Approach

The study aimed to understand trusted sources of COVID-19 vaccine information among hesitant vaccine adopters. Data were collected using an online survey to capture quantitative and qualitative data simultaneously [17,18,19,20,21,22,23]. All study materials and procedures were approved by the University of Arkansas for Medical Sciences Institutional Review Board (IRB# 262645) in July 2021.

### 2.2. Participant Recruitment

Potential respondents were recruited in Arkansas between 22 April 2021 and 6 July 2021, from churches, clinics, and community events where the COVID-19 vaccine was being administered. Vaccine sites were selected through our community-engaged collaborative, which is comprised of both researchers and programmatic networks working to implement a COVID-19 Response Strategy to Reduce Health Disparities [24]. Persons over the age of 18 who could read English, Spanish, or Marshallese were invited to participate in the survey. Potential respondents were approached by study staff and provided a sterilized iPad or a study flyer with the QR code link to the survey to complete on their own electronic device. COVID-19 vaccines were provided to all, including those who chose not to participate in the survey.

### 2.3. Consent and Remuneration

Respondent consent and survey responses were captured using REDCap [25]. REDCap is a widely used web-based software created for research data capture and management [25]. Those who completed the survey and provided contact information were entered into a raffle. The raffle consisted of an opportunity to win a $100 Wal-Mart gift card. One $100 Wal-Mart gift card was given out every day that surveys were completed. 

### 2.4. Data Collection

Survey responses were obtained while respondents waited the required 15 min of observation after receiving their COVID-19 vaccine dose. The survey took approximately 10 min to complete, and the survey was made available to respondents in their preferred language (i.e., English, Spanish, or Marshallese). Bilingual study staff translated text responses for open-ended survey items provided in Spanish or Marshallese to English. 

### 2.5. Instruments

Survey items were selected from validated sources that are part of the PhenX Toolkit [26]. The survey captured socioeconomic demographics, including age, sex, race/ethnicity, education, marital status, and employment status using questions from the Behavioral Risk Factor Survey [27]. Vaccine hesitancy was assessed using a single-item measure of general vaccine hesitancy that we modified [11,28]. Respondents were asked, “Thinking specifically about the COVID-19 vaccine, how hesitant were you about getting vaccinated?” To answer, respondents selected one of these options: “Not at all hesitant”, “A little hesitant”, “Somewhat hesitant”, “Very hesitant”, and “Prefer not to answer”. To understand trusted sources of information among hesitant adopters, respondents were asked the open-ended question, “When deciding whether or not to get the vaccine, what sources of information did you trust the most?” To determine what sources of information they were likely to turn to about the COVID-19 vaccine, participants were asked to select the likelihood they would rely on “a doctor, nurse, or other health care provider”, “family or friends”, “The Centers for Disease Control and Prevention, also known as the CDC”, “Your state or local public health department”, “A pharmacist”, “A religious leader such as minster, pastor, priest, or rabbi”, and “Social media (e.g., Facebook, Twitter, YouTube, WhatsApp)”. Respondents could answer by selecting from response options for each source that included “Very likely”, “Somewhat likely”, “Not too likely”, “Not at all likely”, and “Prefer not to answer”. 

### 2.6. Study Sample

A total of 1475 valid responses to the survey were collected between 22 April 2021 and 6 July 6 2021. Of those, 1452 provided a response to the question on COVID-19 vaccine hesitancy. A total of 867 respondents reported being hesitant when getting their COVID-19 vaccination. These “hesitant adopters” comprise the analytic sample with participants: “very hesitant (*n* = 150)”, “somewhat hesitant (*n* = 269)”, or “a little hesitant (*n* = 448)”. Respondents who selected “prefer not to answer” were excluded. 

### 2.7. Data Analysis

Respondent characteristics and the likelihood of respondents to turn to specific sources of information about the COVID-19 vaccine were tabulated and provided as descriptive summaries. To analyze open-ended items, a qualitative descriptive approach was used to summarize and synthesize respondents’ experiences and perceptions as well as the meanings that respondents attributed to them [29]. MAXQDA 2020 was used to analyze and categorize data into primary themes [30]. Three researchers with qualitative expertise reviewed all responses and developed a codebook with emergent primary themes and subthemes. Data segments were coded, the first author developed the categories, and two additional qualitative researchers performed confirmation-coding analysis. As codes were refined, the codebook was revised three times. The research team critically reviewed each analysis summary to ensure that the data and illustrative excerpts were extracted to the appropriate thematic domain and to ensure analytic rigor and reliability. Any divergences in data interpretation were discussed by the research team and resolved via consensus. The most illustrative quotes were identified under each thematic domain. Many statements are interrelated and expressed within the same sentence, and quotes are presented within the themes they represent best. After codes were finalized, MAXQDA was used to generate the frequency of each primary code among 867 responses. The frequency of each theme is presented.

## 3. Results

Table 1 presents descriptive statistics for age, sex, race/ethnicity, education, marital status, and employment status. The majority of respondents were female (60.21%) and between the ages of 18 and 44 years old (71.97%). Respondents were diverse with most identifying as White (44.54%) or Hispanic/Latinx (32.55%); 8.46% identified as Native Hawaiian or Pacific Islander, 6.46% as Black/African American, 4.70% as Asian, 2.35% as Multiracial, and 0.94% as American Indian/Alaska Native. Just over half of respondents said they were not married (52.63%). A majority of respondents reported they had some college or a four-year degree or more (61.67%). Half of the respondents said they were employed full-time (50.61%). The sample is diverse by race and ethnicity; however, the sample is over-representative of women, and a disproportionate amount of respondents reported a four-year degree (37.86%) than the overall state population reporting a four-year degree (32.10%) in the state of Arkansas [31].

Within the quantitative options, hesitant adopters reported the likelihood they would turn to seven sources for information about the COVID-19 vaccine (Table 2). Almost two-thirds of respondents (64.88%) said they were ‘very likely’ to turn to a health care provider for information about the vaccine, and over half (51.87%) were ‘very likely’ to use the CDC for vaccination information. Respondents were also ‘very likely’ to turn to family (42.64%), the health department (40.88%), or a pharmacist (37.95%) for COVID-19 vaccine information. Some respondents (18.86%) said they were ‘very likely’ to turn to a religious leader for COVID-19 vaccination information. Finally, some respondents (15%) reported they were ‘very likely’ to turn to social media for COVID-19 vaccine information.

**Figure 1:**
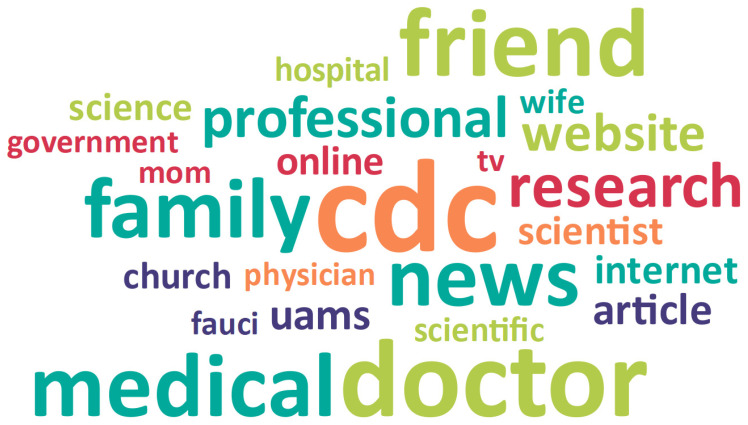
Word Cloud representation of trusted sources of information about COVID-19 vaccine.

Within the qualitative data, hesitant adopters reported multiple sources of trusted information about the COVID-19 vaccine. Trusted sources of vaccine information can be grouped into four emergent subthemes: (1) Health care/Medical science (50.2%), (2) Personal relationships (21.8%), 3) News and social media (16.4%), and 4) Individual/Myself (4.2%). These categories represent 92.5% of the coded responses related to trusted sources of information about the COVID-19 vaccine. Some hesitant adopters reported they did not trust any sources of information (7.5%), describing a lack of trust in traditional sources of information like the mainstream news media or government. Table 3 presents definitions and examples for each emergent subtheme, and Figure 1 provides a Word Cloud representation of respondents’ trusted sources of information. 

### 3.1. Trusted Sources of Information

#### 3.1.1. Health Care/Medical Science

Over half of respondents (50.2%) described health care and medical science sources as their trusted source of information about the COVID-19 vaccine. Within the primary theme of health care providers/medical science, respondents reported two specific sources they trusted for vaccination information: (1) Health care providers (36.3%) and (2) Public health organizations and leaders (13.9%). 

##### Health Care Providers

Respondents reported a range of health care providers they considered a trusted source of COVID-19 vaccine information, including academic medicine and health science research, local medical institutions, and health care providers they know personally. 

When describing academic medicine and health science research, most respondents said “science”, “scientists”, or “research”. One respondent said, “I trusted the scientists that they would not have put it on the market if it wasn’t safe”. Another reported, “The sources of medical information that I trust the most would be scientists and medical staff that know more information about this virus”. Other respondents said they trusted “recent research articles”, “legitimate reports of efficacy”, and “scientific papers in recognized journals”.

Respondents also reported local medical institutions and hospitals as trusted sources of information about the COVID-19 vaccine, stating, “hospital” or “UAMS [University of Arkansas for Medical Sciences, the state’s only academic medical center]”. Respondents reported, “Actual hospitals”, “hospital websites”, and “information directly from hospitals”. One respondent said, “I trusted the people I called at UAMS”. Other respondents stated they trusted “employees” and “information given at the local academic medical center”. 

Respondents reported health care providers as a trusted source of information, with most stating “doctors”, “nurses”, or “doctors and medical field professionals”. Respondents also reported health care providers they know personally (i.e., primary care physician, family members, friends) as trusted sources of information about the COVID-19 vaccine. One respondent said, “My personal doctor. He confirmed that it was a safe and best way to go about the virus”. Another respondent said, “I talked to my doctor and he highly recommended it”. Other respondents reported, “My primary care doctor and specialized doctor”, “medical doctors, pharmacists, and nurses”, and “medical professionals I know and trust”. Other respondents stated family members or friends in the health care field were trusted sources of COVID-19 vaccine information. A respondent said, “My mother who was a nurse for many years, her friends who have studied viruses. Those who work in the medical fields and that I know personally”. Other respondents reported, “Medical doctor and trusted health care friends”, “friends and family in the medical field”, and “friends with medical expertise”. Other respondents said, “My wife, she’s a nurse”, “nurse in my family”, and “my mother, a nurse”.

##### Public Health Organizations and Leaders

When describing public health organizations and leaders, respondents consistently reported the CDC, Arkansas Department of Health, and Dr. Anthony Fauci [Director of National Institute of Allergy and Infectious Diseases] as trusted sources of COVID-19 vaccine information. The majority of respondents simply stated, “CDC”, “health department”, or “Dr. Fauci” when discussing public health organizations and leaders as trusted sources. One respondent said, “Dr. Fauci and other scientist of world leaders pushed me to do this”. Another respondent echoed this: “Dr. Fauci. I trust him more than any other public figure and value his years of experience and service to our country”. Other respondents stated, “Information from the CDC”, “CDC and any department of health”, and “the CDC has provided very great information about how the vaccine works”.

#### 3.1.2. Personal Relationships

A significant number of respondents (21.8%) described personal relationships as their trusted source of information about the COVID-19 vaccine in their open-ended responses. A respondent explained, “I have considerably more trust in the educated opinions of people I know”. Within this primary theme, respondents reported four specific sources they trusted for vaccination information: (1) Family and friends (16.7%), (2) Vaccinated individuals (2.0%), (3) Religious institutions and leaders (2.6%), and (4) Employers and coworkers (0.5%).

##### Family and Friends

When describing family and friends as trusted sources of information, respondents typically said, “my family” or “my friends”. A respondent reported, “Information from my parents”. Another said, “I got my information from family and friends” and “I mostly trusted my parents”. Others said, “My sister and my husband” and “my mom”. Merging into another theme, respondents explained that family and friends who had already received their vaccine were their trusted source of information. One respondent reported, “Family members and friends who have been vaccinated. It was helpful to hear a variety of symptoms and experience with the vaccine”. Another respondent said, “Previous family members receiving it and them being okay with it”. Others said, “Friends who had taken the vaccine already” and “I asked friends and family that have gotten the vaccine about the symptoms”.

##### Vaccinated Individuals

Some respondents discussed vaccinated individuals generally as a trusted source of information. A respondent said, “I really trusted people who had already gotten the vaccine”. Another respondent stated, “People that have gotten the vaccine before me”. Respondents reported, “Personal experiences of people I know”, “Other people’s experiences”, and “In the experiences of those who have already gotten vaccinated”. One respondent reported, “Just observing the ones that had it done” as a trusted source of information about the COVID-19 vaccine.

##### Religious Institutions and Leaders

While the proportion was lower than many other responses, respondents also identified religious institutions and leaders as trusted sources of information. When discussing religious institutions and leaders, respondents said “the church” or “my church”. One respondent reported that “the information that they gave us at the church” was a trusted source. Another respondent said, “My church sent out an email with some information which also helped me tremendously”. Others reported, “Trusted ecclesiastical leaders”, “The priest of the church”, and “the information that the church is giving” as trusted sources. 

##### Employer and Coworkers

A small number of respondents (0.5%) reported their employer and coworkers as trusted sources of COVID-19 vaccine information. One respondent said, “My job provided information”. Another respondent said, “Updated information provided by JB Hunt”. Other respondents reported “Work” or “Coworkers” as trusted sources of vaccination information.

#### 3.1.3. News and Social Media

Some respondents (16.4%) reported news and social media as trusted sources of information about the COVID-19 vaccine in their open-ended responses. Within this theme, respondents reported two specific sources: (1) News media (9.1%) and (2) Internet (7.3%). 

##### News Media

When describing news media sources they trusted for information, respondents typically said “the news”, “newspapers”, “creditable news”, or “the radio”. Other respondents reported, “MSNBC and CNN”, “PBS”, “Local news”, and “Fox News” as trusted sources of vaccine information.

##### Internet

Respondents also reported online sources they trusted for COVID-19 vaccine information. One respondent said, “Science websites”. Others said, “Medical online sources”, “official medical websites”, and “credible online sources such as CDC.gov”. Another respondent stated they trusted “Independent leftist news sources on YouTube, Instagram, and Facebook”. One respondent reported, “Local news website and social media page that links to healthcare pages” as trusted sources. Respondents also reported simply, “online sources”, “websites”, “Facebook”, “Google”, and the “internet”.

#### 3.1.4. Individual/Myself

A few respondents (4.2%) reported themselves as a trusted source of COVID-19 information in their open-ended responses. Within this theme, respondents discussed two specific sources they trusted for information: (1) Trusted my gut and (2) Did my own research.

##### Trusted My Gut

A respondent reported, “I trusted my gut and intuition” for vaccine information. Another respondent said, “Really just trusting my gut”. Other respondents said, “Just myself”, “Me”, or “my gut” were trusted sources. 

##### Did My Own Research

A few respondents said they trusted their own research as a trusted source of information about the COVID-19 vaccine. One respondent reported, “I read some articles on the Pfizer vaccine”. Another respondent said, “Doing research on it” and “cross-referencing multiple sites and facts”. One respondent stated, “I conducted my own research”, and another respondent reported, “I used Google to find trustworthy news sources on the various vaccines to determine which one to get”.

### 3.2. Do Not Trust Any Sources of Information

Almost one in ten respondents (7.5%) discussed distrust or a lack of trust in sources for information about the COVID-19 vaccine in their open-ended responses. Within this primary theme, respondents reported two subthemes: (1) Do not trust any sources and (2) Lack of trust in traditional sources. 

#### 3.2.1. Do Not Trust Any Sources 

One respondent reported, “None. So much conflicting information”. Another respondent said, “None of it. I don’t believe in the vaccine at all. It is just an untested glorified flu shot to me”. A respondent stated, “All of this was bull shit. Everything contradicted each other or even themselves. I have zero faith in any of this”. Other respondents said, “Not any”, “None at all”, and “Nothing”.

#### 3.2.2. Lack of Trust in Traditional Sources

A few respondents reported they lacked trust in traditional sources of information such as the news media or government. A respondent explained, “I don’t feel that there are many unbiased media sources”. Another respondent said, “No sources as I don’t trust the news or government”. Others said, “Not the news since the double or the single shot are more or less experimental”, and “Definitely not the media”. Another respondent said, “Allow me to answer the opposite question: I have little to no trust in mass media”. 

## 4. Discussion

This study documents the trusted sources of information among hesitant adopters as well as a lack of trust in traditional sources of information about the COVID-19 vaccine using quantitative and qualitative methods. Respondents reported health care and medical science sources as their trusted source of information about the COVID-19 vaccine as the most frequent option in both the qualitative and quantitative data. State and federal public health organizations and leaders were also reported as trusted sources of COVID-19 vaccine information. These findings are consistent with prior research that documents high levels of trust in health care providers [32] and the CDC [33,34,35] as sources of general COVID-19 information. This data also supports previous findings of high levels of trust for scientists, health departments, and health professionals as sources of information about the COVID-19 vaccine [14,16,36]. While some studies suggest that there has been a decline in trust of health care providers and organizations as sources of information related to COVID-19 [35,37,38,39], our study and others [33,34,35] document that health care providers and organizations are the greatest sources of trusted information regarding COVID-19, including among hesitant adopters.

Respondents described personal relationships—family and friends, religious institutions and leaders, employers, and coworkers—as trusted sources of vaccine information. Respondents described how personal testimonies from vaccinated individuals about their experiences were also a trusted source of information. This notable finding demonstrates the importance of personal testimonies of vaccination experiences that could inform public health messaging campaigns to increase vaccine uptake. This finding is consistent with research that has documented social relationships as a primary influence of vaccination uptake [40]. The qualitative responses demonstrated that health care providers who respondents knew personally were an important trusted source of information. These sources were described as having both expertise and a valued social connection. This overlap of personal relationships with specific expertise may increase perceptions of trustworthiness.

A few respondents reported the news media and internet as trusted sources of COVID-19 vaccine information [35,37,38,39]. While historically news media such as national and local television, newspapers, and radio have been important sources of health information [37,41,42], a 2020 study (*n* = 10,139) reported that less than half of US adults (49%) said the news media’s coverage of COVID-19 has been largely accurate [43,44]. Our study results are consistent with previous findings showing that the news media is often seen as less trustworthy when compared to other sources of information about COVID-19 [34,35,37] and the COVID-19 vaccine specifically [14,36].

Some respondents expressed a distrust of all sources of information about the COVID-19 vaccine, even though they had received the vaccine. Among those who cited a lack of trust, several respondents described not trusting traditional sources of information, such as the mainstream media and government. These findings are consistent with previous studies documenting a decline in Americans’ trust in COVID-19 information provided by the news media and federal and state governments [38,39,43].

### 4.1. Strengths and Limitations

The study is not without limitations. Survey respondents were recruited as they waited the required fifteen minutes after receiving their COVID-19 vaccine dose, so this non-random sample may not be representative of the general populations of Arkansas or the US. The study sample size was not pre-determined, and all people who participated in the survey were included. Respondents who reported some level of vaccine hesitancy and who got the COVID-19 vaccine comprised the study sample. Generalizability is improved because the sample is large and diverse. It is not known to what degree respondents were exposed to different modes of information, and this limits comparative analysis. Open-ended questions allowed respondents to provide their typed responses anonymously and to describe their perceptions of trusted sources of COVID-19 vaccine information in their own words; however, the research team lacked the opportunity to probe for clarification of respondents’ typed responses. Despite these limitations, this study documents trusted sources of information among hesitant adopters and provides important information that can be used to improve health messaging and increase vaccine uptake among those who are hesitant.

### 4.2. Recommendations and Implications for Practice

This study documents trust in health care and personal relationships as sources of information about the COVID-19 vaccine. Respondents reported being very likely to turn to a health care provider, the CDC, and family as sources of information when deciding if they will get vaccinated. In particular, respondents said that hearing about the experiences of people who were vaccinated was a trusted source of COVID-19 vaccine information. Personal testimony from those who are vaccinated, such as local health care providers or community members, could be important to marketing campaigns intended to increase vaccine uptake. Future research should examine trusted sources among hesitant non-vaccinated individuals to identify additional factors that may encourage vaccine uptake.

## 5. Conclusions

This study makes an important contribution to the literature as the first mixed-methods study on trusted sources of COVID-19 vaccine information among hesitant adopters. The study documents trusted sources of vaccine information among hesitant adopters, such as health care providers, family and friends, and vaccinated individuals. The study also documents a lack of trust in traditional sources of information including the news media and the government. These findings provide important insights about respondents’ trusted sources of COVID-19 vaccine information that can inform future public health messaging campaigns intended to increase vaccine uptake among hesitant adopters.

## Figures and Tables

**Table 1:** Descriptive characteristics of recently vaccinated Arkansans (*n* = 867).

	Frequency	% or $\bar{x}$
Age	867	37.21
18–24	178	20.53%
25–34	219	25.26%
35–44	227	26.18%
45–54	136	15.69%
55–64	84	9.69%
65+	23	2.65%
Sex	862	
Female	519	60.21%
Male	343	39.79%
Race/Ethnicity	851	
American Indian/Alaska Native	8	0.94%
Asian	40	4.70%
Black/African American	55	6.46%
Native Hawaiian or Pacific Islander	72	8.46%
White	379	44.54%
Hispanic/Latinx	277	32.55%
Multiracial	20	2.35%
Education	840	
Less than High School	110	13.10%
High School or GED	212	25.24%
Some College	200	23.81%
Four-Year Degree or More	318	37.86%
Marital Status	838	
Married	397	47.37%
Not Married	441	52.63%
Employment Status	816	
Full Time	413	50.61%
Part Time	82	10.05%
Other	321	39.34%
COVID-19 Vaccine Hesitancy	867	
A Little Hesitant	448	51.67%
Somewhat Hesitant	269	31.03%
Very Hesitant	150	17.30%

**Table 2:** Likelihood of turning to information sources when deciding whether to get a COVID-19 vaccine among hesitant adopters.

	Very Likely	Somewhat Likely	Not too Likely	Not at All Likely	PNA *
Health Care Provider	558 (64.88%)	210 (24.42%)	54 (6.28%)	23 (2.67%)	15 (1.74%)
CDC	444 (51.87%)	251 (29.32%)	89 (10.40%)	55 (6.43%)	17 (1.99%)
Family	368 (42.64%)	318 (36.85%)	130 (15.06%)	35 (4.06%)	12 (1.39%)
Health Department	352 (40.88%)	331 (38.44%)	106 (12.31%)	56 (6.50%)	16 (1.86%)
Pharmacist	326 (37.95%)	274 (31.90%)	164 (19.09%)	77 (8.96%)	18 (2.10%)
Religious Leader	162 (18.86%)	176 (20.49%)	173 (20.14%)	320 (37.25%)	28 (3.26%)
Social Media	129 (15.00%)	153 (17.79%)	222 (25.81%)	337 (39.19%)	19 (2.21%)

* Note: Prefer Not to Answer (PNA).

**Table 3:** Primary themes and emergent subthemes with definitions and examples.

Primary Themes	Subthemes
Trusted sources of information (92.5%)	*Health care/Medical science (50.2%)* Health care providers Academic medicine and health science research Local medical institutions Health care providers they know personally (i.e., PCP, family member, friends) Public health organizations and leaders CDC Arkansas Department of Health Dr. Fauci
	*Personal relationships (21.8%)* Family and friends Vaccinated individuals Religious institutions and leaders Employer and coworkers
	*News and social media (16.4%)* News media Internet
	*Individual/Myself (4.2%)* Trusted my gut Did my own research
Do not trust any sources of information (7.5%)	Do not trust any sources Lack of trust in traditional sources News media Government

## Data Availability

The deidentified data underlying the results presented in this study may be made available upon reasonable request from the corresponding author, Dr. Pearl A. McElfish, at pamcelfish@uams.edu. The data are not publicly available in accordance with funding requirements and participant privacy.
